# Transcriptional impairment of *β*-catenin/E-cadherin complex is not associated with *β*-catenin mutations in colorectal carcinomas

**DOI:** 10.1038/sj.bjc.6600706

**Published:** 2003-01-28

**Authors:** G A Garinis, N E Spanakis, P G Menounos, E N Manolis, G Peros

**Affiliations:** 1 Nursing Military Academy, Laboratory of Research, Athens, Greece; 2 Nikaia Hospital, Department of Surgery, Athens, Greece; 3 Department of Anatomy and Histology, School of Nursing, University of Athens, Athens, Greece

**Keywords:** colorectal, *β*-catenin, mRNA, mutations

## Abstract

We report the absence of *β*-catenin mutations in 63 sporadic colorectal carcinomas (SCRCs) with demonstrated decreased *β*-catenin and E-cadherin mRNA expression and E-cadherin protein expression in a subset of carcinomas examined, suggesting that *β*-catenin mutations are an extremely rare phenomenon in SCRCs and are not responsible for the transcriptional impairment of the *β*-catenin/E-cadherin adhesion complex observed in these tumours.

*β*-catenin is a 92-kDa multifunctional protein that, when localised in the membrane, links the intracellular part of the E-cadherin complex to the actin cytoskeleton, a step critical to morphogenesis and the maintenance of tissue architecture (Aberle *et al*, 1994). Alternatively, through Wnt signalling-mediated stabilisation, *β*-catenin may act as a downstream transcriptional transactivator of several target genes (Barth *et al*, 1997). Alterations in *β*-catenin protein expression levels and genetic rearrangements located in *β*-catenin exon 3 have been shown to contribute to the malignant character of various carcinomas and are likely to affect both intercellular adhesion and signal transduction, which are believed to be two independent functions of the *β*-catenin protein (Fujimori *et al*, 2001). The rarity of mutations within *β*-catenin exon 3 has been previously documented in ulcerative colitis-related neoplastic progression (Nilbert *et al*, 2001) and in rectal cancer (Aust *et al*, 2002) but, to the best of our knowledge, not in SCRCs. In fact, the variable immunohistochemical studies performed suggest that the observed accumulation in *β*-catenin protein is probably because of genomic alterations in *β*-catenin coding regions and, in particular, *β*-catenin exon 3. This study rules out significantly this possibility, indicating that other mechanisms (i.e. those that might be involved in the regulation of the *β*-catenin mRNA level) may play a role in the observed protein accumulation. Furthermore, the effect of these genetic alterations on the transcriptional activity of the *β*-catenin/E-cadherin adhesion complex is not yet known. Within this context, we investigated the possible association of *β*-catenin mutations in exon 3 with the *β*-catenin/E-cadherin adhesion complex transcriptional activity by determining their mRNA levels and the levels of E-cadherin protein expression in a subset of these carcinomas.

## MATERIALS AND METHODS

### Tumour tissues and control samples

A total of 63 sporadic colorectal adenocarcinomas and adjacent normal tissues at least 2 cm from the distal negative margin of the tumour were obtained from surgically treated patients at Nikaia Hospital, Athens, Greece. None of the patients received irradiation preoperatively.

### PCR-single-strand conformation polymorphism (SSCP) and *β*-catenin exon 3 genomic rearrangements

**Figure 1:**
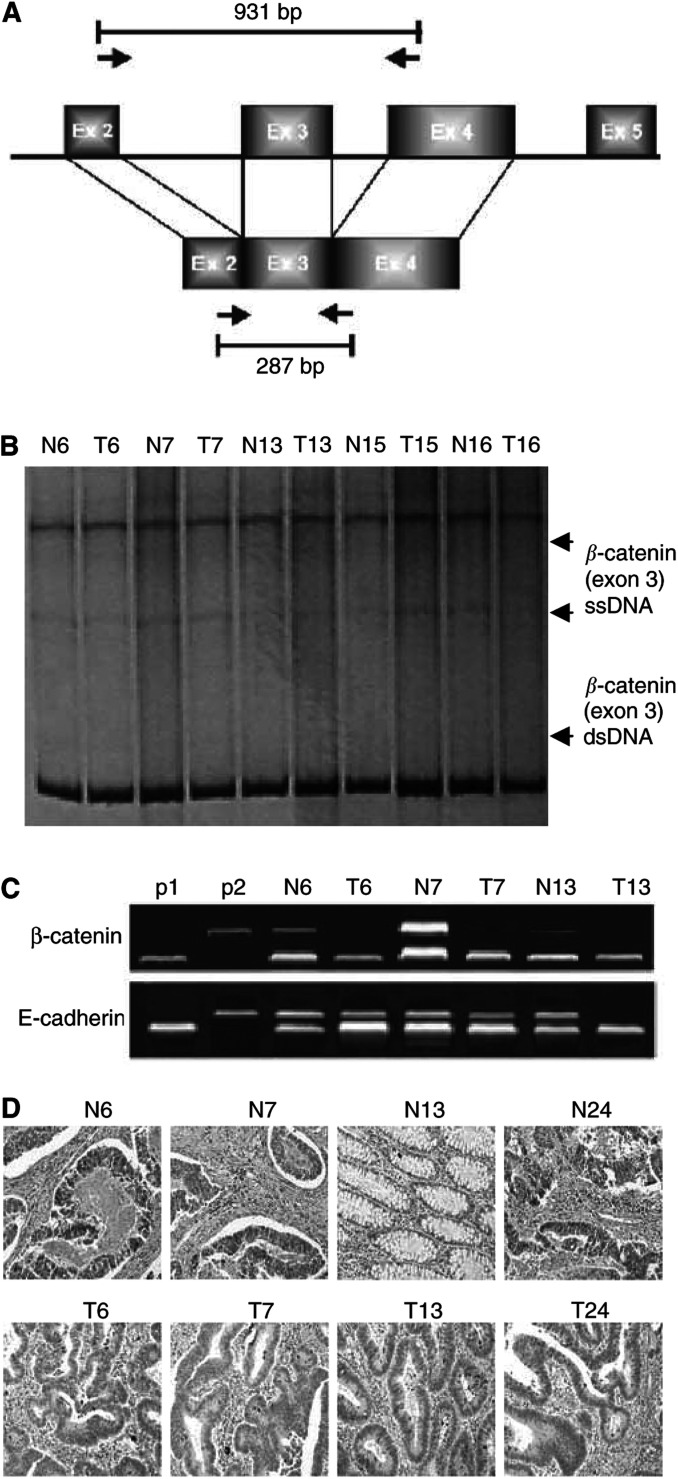
(**A**) Schematic representation of the primer set used to amplify genomic DNA and cDNA of the *β*-catenin gene. (**B**) SSCP analysis of the *β*-catenin exon 3. Arrowheads indicate single-stranded (ss) and double-stranded (ds) DNA. (**C**) RT–PCR analysis of the *β*-catenin and E-cadherin mRNA levels. P1 and P2 represent 18S rRNA and *β-*catenin or E-cadherin positive controls, respectively. As an example, samples 6, 7 and 13 demonstrated a decrease of mRNA expression in both E-cadherin and *β*-catenin followed by a parallel decrease in E-cadherin protein expression as depicted in **D**. (**D**) E-cadherin immunohistochemical detection in SCRCs in tumour (T) and adjacent normal tissue (N) sections. Samples N6, N7, N13 and N24 demonstrated a uniform expression of E-cadherin protein along intercellular borders in normal colonic mucosa. Samples T6, T7, T13 and T24 exhibited a decreased and diffused cytoplasmic staining with occasional intensified immunoreactivity at the luminal surface.

cDNA from 63 tumour specimens was amplified for SSCP screening to detect *β*-catenin nucleotide substitutions as previously described (Iwao *et al*, 1998). The same primer set was used to amplify genomic DNA corresponding to DNA sequences in exons 2 and 4 to detect genomic rearrangements involving exon 3 and for automated sequencing (Figure 1A

).

### Multiplex RT–PCR analysis

Owing to sample unavailability, RT–PCR analysis for *β*-catenin and E-cadherin was carried out in a subset of 37 SCRCs from the same series. cDNA primers and amplification conditions for *β*-catenin and E-cadherin have been previously described (Oda *et al*, 1994; Iwao *et al*, 1998). 18S rRNA primers were used to amplify the invariant endogenous control (Quantum RT–PCR, Ambion, USA). A two-fold increase or decrease was considered as the cutoff value. Since identical multiplex quantitative reactions may still exhibit some degree of variation, all reactions were performed in duplicates. The level of the product from *β*-catenin and/or E-cadherin gene was normalised against 18S rRNA as follows: all raw data were first transformed to a logarithmic scale. The latter makes variation of band intensities and ratios of band intensities independent of the absolute magnitude and evens out highly skewed distributions. Following log transformation, we divided, for each sample, the signal obtained for the E-cadherin-specific amplicon by the signal obtained for the 18S rRNA amplicon. This yielded a corrected relative log ratio that was the weighted average from each pair of duplicate PCR reactions for each gene-specific product in each sample. Log ratio values were then compared between samples for an estimate of the relative expression of target RNA in these samples. Negative and positive controls for *β*-catenin, E-cadherin and 18S rRNA amplicons were included in each amplification.

### Immunohistochemistry (IHC)

E-cadherin immunohistochemical analysis was performed with the antibody (Ab) H-108 (class: rabbit polyclonal; epitope: residues 600–707; Santa Cruz, CA, USA) in formalin-fixed paraffin-embedded tissue sections from the same subset of 37 SCRCs as previously described (Ilyas *et al*, 1997). The extent of staining was compared to normal paired tissue sections and IHC evaluation was performed as follows: grade 0: 0% of stained cells; grade 1: 1–30%; grade 2: 31–50%; grade 3: >50%. Incubating paraffin sections in the absence of the primary antibody tested the specificity of E-cadherin primary Ab H-108.

### Statistical methods

Statistical analysis was performed using the two-sided Kruskal–Wallis test validated by Fisher's exact test where appropriate. Statistical significance was set at *P*<0.03.

## RESULTS AND DISCUSSION

The clinical and pathological features for the subset of 37 SCRC patients (20 males and 17 females, mean age: 67.3 years; range: 53–84 years) are shown in Table 1

**Table 1:** *β*-catenin and E-cadherin mRNA expression in association with patients' clinicopathological features and E-cadherin protein immunohistochemical detection

**(A)**									
	***β*-catenin mRNA**			**E-cadherin mRNA **			**E-cadherin IHC**		
	**ND *(n=7)***	**− *(n=24)***	***+ (n=6)***	**ND *(n=6)***	**− *(n=25)***	***+ (n=6)***	**I *(n=21)***	**II *(n=10)***	**III *(n=6)***
**Gender**									
Females	2	11	2	3	12	2	6	4	3
Males	5	13	4	3	13	4	15	6	3
									
**Location**									
Right	2	8	2	2	8	3	8	6	2
Left	2	7	2	1	7	2	10	3	3
Rectum	3	9	2	3	10	1	3	1	1
									
**LN**									
No	5	4	3	3	3	5	1	4	2
Yes	2	20	3	3	22	1	20	6	3
									
	**(*P*=0.018)**	**(*P*=0.001)**	**(*P*=0.012)**						
**Grade**									
Well/mod	2	9	2	1	13	1	12	1	2
Poor	5	15	4	5	12	5	9	9	4
									
**Stage**									
A	1	0	0	0	1	0	1	0	3
B1-B2	2	8	2	1	6	4	4	4	2
C1-C2	4	16	4	5	18	2	16	6	1
									
**Tumour Size**									
0–3	3	4	4	2	3	3	11	3	2
3.1–6	4	20	2	3	18	2	7	7	2
6.1–11	0	2	0	0	2	1	4	0	2
									
(**B**)									
	***β*-catenin mRNA**			**E-cadherin mRNA**					
	**ND**	**− **	**+**	**ND**	**− **	**+**			
*E-cadherin IHC*									
I	3	17	4	2	23	0			
	**(*P*=0.003)**	**(*P*=0.001)**							
II	3	6	3	2	4	4			
III	0	1	2	0	1	1			

ND=no difference in mRNA expression between normal and tumour sample; LN=lymph node metastasis; mod=moderate; A-C2=Astler–Coller's classification.

(A). The SSCP screening on *β*-catenin exon 3 did not demonstrate any nucleotide substitutions or polymorphisms in all 63 SCRCs examined (Figure 1B). Similarly, PCR-amplified experiments using genomic DNA with the same primer set corresponding to DNA sequences in exons 2 and 4 did not detect any PCR products smaller than the expected 931 base-pair (bp) product (Figure 1A). These results indicate that *β*-catenin mutations and/or splicing changes comprise an infrequent phenomenon in SCRCs, revealing the possibility that various regulatory mechanisms acting on *β*-catenin mRNA expression may determine its oncogenic potential.

It was surprising that subsequent RT–PCR analysis in a subset of 37 SCRCs demonstrated a more than two-fold decreased expression of *β*-catenin mRNA in 24 out of 37 (65%) of the carcinomas examined ( Table 1(A), Figure 1C). Few studies have focused on the mutational analysis of the *β*-catenin gene demonstrating an altered IHC expression of *β*-catenin without genetic alterations (Rimm *et al*, 1999; Bilim *et al*, 2000). However, none of these studies confirmed *β*-catenin expression at the mRNA level and there might be a possibility that the reported changes in antigen availability have occurred as a result of post-translational modification. The decreased expression of *β*-catenin at the mRNA level may indicate a high turnover that keeps up the steady-state levels of *β*-catenin expression at the protein level. Alternatively, it may point out a *β*-catenin epigenetically associated transcriptional silencing similar to that previously reported for E-cadherin inactivation in SCRCs (Garinis *et al*, 2002). Therefore, the possibility remains that for *β*-catenin, promoter hypermethylation could be as critical for, and possibly as frequent, in SCRC evolution as mutations in critical coding regions.

A paralleled decreased expression of E-cadherin mRNA was demonstrated in 25 out of 37 of the carcinomas (68%), and this was followed by a comparable decrease of E-cadherin IHC expression (grade I) in 21 out of 37 (57%) of SCRCs ( Table 1a, Figures 1C and D). E-cadherin IHC expression (grade I) was significantly associated with both *β*-catenin and E-cadherin mRNA expression profiles in 17 out of 37 (42.5%) and 23out of 37 (62%), respectively ( Table 1(B)). The fact that *β*-catenin has two different functions, nuclear signalling and cadherin-mediated adhesion at the plasma membrane, raises the possibility that cadherins could transduce a signal to the nucleus via *β*-catenin. In addition, recent data suggest that in the cytosol, *β*-catenin may bind to GSK-3*β* (Wnt regulatory pathway), APC, and Axin, while in the nucleus binding to TCF leads to promotion of gene transcription (Alexander *et al*, 2002). The Wnt transduction pathway induces the nuclear translocation of *β*-catenin and has a major role in cell fate determination. Tight somatic regulation of this signal is essential, and uncontrolled accumulation of *β*-catenin in the nucleus may lead to tumour initiation in the adult organism. Kielman *et al*, (2002) have provided genetic and molecular evidence that the ability and sensitivity of embryonic stem cells to differentiate into the three germ layers is inhibited by increased dosages of *β*-catenin made available for Wnt signalling by specific APC defects. Furthermore, *β*-catenin may participate in ubiquitination and subsequent degradation pathways (Alexander *et al*, 2002). All data presented above point out *β*-catenin as a major sensor of an array of signals and imply that differentiation defects in tissue homeostasis are likely to underlie tumorigenesis because of inactivation of the APC/*β*-catenin signalling pathway. Since in this study, loss of mRNA expression in one of the components of this adhesion complex was often paralleled by a similar pattern of transcriptional impairment in the other (i.e. E-cadherin), these results point out an additional possible physiological association between E-cadherin expression and the *β*-catenin signalling pathways described above. However, it is not yet known whether changes in E-cadherin mRNA and/or protein expression levels may affect *β*-catenin signalling and our study cannot rule out the possibility of cytoplasmic *β*-catenin stabilisation at the protein level.

Both decreased *β*-catenin and E-cadherin gene expression were significantly associated with the presence of lymph node metastases (LN) (*P*=0.018) in 20 out of 37 (54%) and in 22 out of 37 (59%) (*P*=0.001) of the carcinomas examined, respectively. Similarly, a significant inverse association was observed between decreased E-cadherin IHC detection (grade I) and LN presence (*P*=0.012) in 20 out of 37 (54%) of these carcinomas. The demonstrated impairment of *β*-catenin/E-cadherin adhesion and its association with LN metastasis ( Table 1) suggests that the inactivation of the E-cadherin/*β*-catenin system through multiple mechanisms and the subsequent suppression of cell-cell adhesiveness may trigger the release of cancer cells from the primary tumour foci and confer invasive properties on the primary tumour mass.

In essence, this study shows that the mutations within *β*-catenin exon 3 are an infrequent event in SCRCs and that *β*-catenin genetic alterations are not responsible for the demonstrated loss of *β*-catenin/E-cadherin transcriptional activity.
